# Identification of therapeutic targets for breast cancer using biological informatics methods

**DOI:** 10.3892/mmr.2015.3565

**Published:** 2015-03-27

**Authors:** XUEJIAN LIU, YONGZHEN MA, WENCHUAN YANG, XIA WU, LIHUA JIANG, XIANGLI CHEN

**Affiliations:** 1 Department of Oncology, The People’s Hospital of Linyi Economic and Technological Development Zone, Linyi, Shandong 276023, P.R. China; 2 Department of Histological Embryology, Shandong Medical College, Linyi, Shandong 276000, P.R. China

**Keywords:** breast cancer, differentially expressed genes, protein-protein interaction network, therapeutic targets

## Abstract

The present study aimed to investigate the modular mechanisms underlying breast cancer and identify potential targets for breast cancer treatment. The differentially expressed genes (DEGs) between breast cancer and normal cells were assessed using microarray data obtained from the Gene Expression Omnibus database. Gene ontology (GO) and pathway enrichment analyses were performed in order to investigate the functions of these DEGs. Subsequently, the protein-protein interaction (PPI) network was constructed using the Cytoscape software. The identified subnetworks were further analyzed using the Molecular Complex Detection plugin. In total, 571 genes (241 upregulated and 330 downregulated genes) were found to be differentially expressed between breast cancer and normal cells. The GO terms significantly enriched by DEGs included cell adhesion, immune response and extracellular region, while the most significant pathways included focal adhesion and complement and coagulation cascade pathways. The PPI network was established with 273 nodes and 718 edges, while fibronectin 1 (FN1, degrees score, 39), interleukin 6 (IL6; degree score, 96) and c-Fos protein (degree score, 32) were identified as the hub proteins in subnetwork 2. These dysregulated genes were found to be involved in the development of breast cancer. The *FN1*, *IL6* and *FOS* genes may therefore be potential targets in the treatment of breast cancer.

## Introduction

Breast cancer is a common type of tumor, which develops in the breast tissue. The majority of breast cancer cases occur in females, which may result from gender-associated characteristics (1) and certain hormones (2). Additional risk factors contributing to breast cancer development include genetics (3), obesity (4) and environmental pollution (5). Breast cancer is one of the leading causes of cancer-associated death amongst females, accounting for ~23% of all cancer cases diagnosed in females (6). In 2008, 458,503 deaths resulted from breast cancer worldwide (7). Although the survival rate (~85%) for breast cancer is higher in Western countries, it is significantly lower in developing countries (6). Therefore, breast cancer is a global health concern.

Significant progress has been achieved in the elucidation of the pathological mechanisms underlying the development of breast cancer (8,9). Certain genes have been identified to be involved in the progression of breast cancer. Breast cancer-specific gene 1 (*BCSG1*), also known as synuclein γ, was demonstrated to be overexpressed in breast tumor tissues and stimulated the metastasis of breast tumor cells (10). The elevated expression levels of nuclear receptor coactivator 3 (ncoa3; *AIB1*) and human epidermal growth factor receptor 2 (*HER2*) in breast cancer cells were demonstrated to contribute to tamoxifen resistance (11). Furthermore, certain significant pathways have also been found to play a crucial role in breast cancer. The *HER2* tyrosine kinase pathway promoted hormone-independent growth and enhanced endocrine resistance in breast cancers (12). In addition, the activity of the Hedgehog signaling pathway in breast cancer cells was found to result in abnormal growth of the mammary duct and may therefore represent a candidate target for breast cancer treatment (13). Progress has been achieved in the elucidation of the mechanisms underlying breast cancer development, contributing towards the development of novel therapeutic methods. However, the present knowledge is insufficient.

In the present study, a biological informatics approach was used to analyze the gene expression profiles in breast cancer cells, while a functional analysis was performed in order to identify differentially expressed genes (DEGs) between breast tumor cells and matched normal tissues. Additionally, a protein-protein interaction (PPI) network was constructed. The present study aimed to generate a systematic perspective to understanding the underlying mechanisms and identifying novel therapeutic targets for breast cancer.

## Materials and methods

### Affymetrix microarray analysis

The array data for GSE26910, were downloaded from the Gene Expression Omnibus (GEO; http://www.ncbi.nlm.nih.gov/geo/) database, as reported by Planche *et al* (14). A total of 24 samples were used in the development of the Affymetrix microarray data. The expression profiles analyzed in this work were derived from 12 samples, including six samples of stroma surrounding invasive primary breast tumors and six samples of normal stroma breast tissues. The raw CEL data and annotation files were downloaded based on the GPL570 platform (Affymetrix Human Genome U133 Plus 2.0 Array; Affymetrix, Inc., Santa Clara, CA, USA) for further analysis.

### Data processing and DEG analysis

The raw expression data were preprocessed using the robust multiarray average (15) algorithm with application of the Affy package (version 1.44.0; Fred Hutchinson Cancer Research Center, Seattle, WA, USA) in the R statistical software (version 3.1.2; Bell Labs, Murray Hill, NJ, USA). When multiple probes corresponded to the same gene, the mean value was calculated as the expression value of that gene.

The DEGs between breast cancer tissues and matched normal tissues were analyzed using the linear models for microarray data (limma) package (version 3.22.1; Fred Hutchinson Cancer Research Center) (16). |log of fold change|>1 and P<0.01 were considered to be the cut-off values for DEG screening.

### Gene ontology (GO) and pathway enrichment analysis

GO is a tool for the unification of biology which collects structured, defined and controlled vocabulary for large scale of gene annotation (17). In addition, the Kyoto Encyclopedia of Genes and Genomes (KEGG; http://www.genome.jp/kegg/) database is used for the classification of correlating gene sets into their respective pathways (18).

In order to analyze the DEGs at a function level, GO annotation and KEGG pathway enrichment analyses for DEGs were performed using the Database for Annotation, Visualization and Integration Discovery (DAVID) software (version 6.7; http://david.abcc.ncifcrf.gov). The DEGs were classified into three GO categories, including molecular function (MF), biological process (BP) and cellular component (CC). P<0.01 was set as the threshold value.

### PPI network construction

Search Tool for the Retrieval of Interacting Genes (STRING), an online database resource that collects comprehensive information of predicted and experimental interactions of proteins (19), was used in the present study. The interactions of protein pairs in the STRING database were displayed using a combined score. The DEGs were mapped into PPI networks and a combined score of >0.5 was set as the cut-off value for significant protein pairs. The PPI network was established using Cytoscape software (version 1.1.1; National Institute of General Medical Sciences, Bethesda, MA, USA) (20) and the hub node was screened according to the degree score (number of neighbors). The subnetworks (nodes >15) were evaluated using the Molecular Complex Detection (MCODE) plugin of Cytoscape (21). Subsequently, the subnetwork functions were assessed by GO and pathway enrichment analyses of the genes involved in the subnetworks using the DAVID online tool.

## Results

### Data processing and DEG analysis

**Figure 1:**
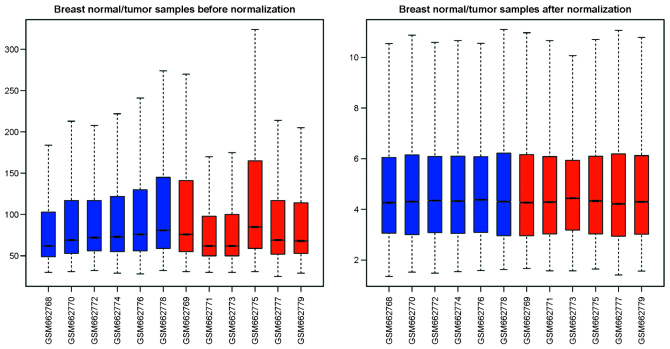
Box plots of results of data normalization. The x coordinate represents the samples and the y-coordinate represents the gene expression values. The midline of the box plot represents the gene expression median and the whiskers represent the interquartile range. Blue box plots, normal breast tissue samples; red box plots, breast cancer tissue samples.

As shown in Fig. 1, the obscuring variations in the raw expression data were normalized following preprocessing. Subsequently, DEG analysis was performed using the limma software package. A total of 571 genes were found to be differentially expressed between breast cancer tissues and normal tissues, among which 241 genes were upregulated and 330 genes were downregulated.

### GO and pathway enrichment analyses

GO and pathway analyses were performed on upregulated and downregulated DEGs, separately. The top five GO terms identified in each of the three GO categories (BP, CC and MF) are shown in Table I. The overrepresented GO terms of upregulated DEGs were associated with cell adhesion, response to wounding, immune response, extracellular region, extracellular matrix, calcium ion binding and actin binding. The downregulated DEGs involved in the three GO categories were as follows: in the BP category, cell surface receptor-linked signal transduction, response to wounding and cell adhesion; in the CC category, plasma membrane, extracellular region and plasma membrane region; and in the MF category, carbohydrate, lipid and polysaccharide binding.

The pathways significantly enriched by the upregulated DEGs included the cytokine-cytokine receptor interaction, focal adhesion and extracellular matrix (ECM)-receptor interaction pathways. By contrast, the two pathways that were enriched by the downregulated DEGs included the axon guidance and complement and coagulation cascade pathways (Table I).

### PPI network analysis

**Figure 2:**
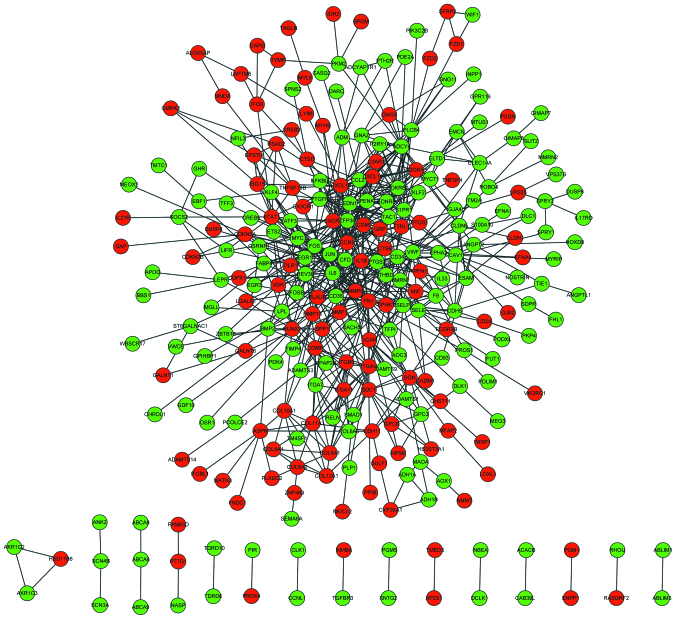
Protein-protein interaction network of differentially expressed genes. In the bottom panel, 20 single protein pairs with combined scores of 0.5 are shown, which presented no interaction with other proteins. Red nodes, upregulated genes; green nodes, downregulated genes.

Based on STRING database analysis, a total of 718 protein pairs with combined scores of >0.5 were identified. As demonstrated in Fig. 2, the PPI network consisted of 273 nodes and 718 edges. The nodes of fibronectin 1 (FN1; degree score, 39), interleukin 6 (IL6; degree score, 96) and c-Fos protein (degree score, 32) were hub proteins in the PPI network.

**Figure 3:**
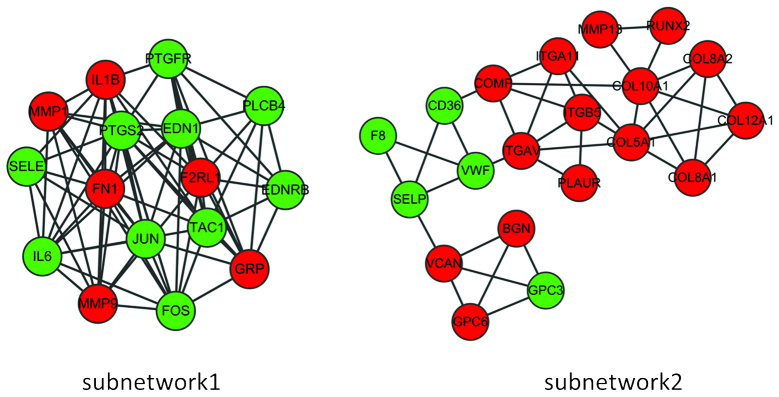
Two subnetworks in the protein-protein interaction network. Red nodes, upregulated genes; green nodes, downregulated genes.

Two subnetworks (subnetworks 1 and 2) with >15 nodes were detected using the MCODE plugin (Fig. 3). The hub proteins FN1, IL6 and FOS were demonstrated to be involved in subnetwork 1. Subnetwork 1 was mainly associated with response to wounding and extracellular region, while the most significant pathway was found to be the Toll-like receptor signaling pathway (Table II). By contrast, subnetwork 2 was associated with cell adhesion, response to wounding, wound healing, glycoprotein binding and calcium ion binding (Table III). In addition, the significant pathways associated with subnetwork 2 were focal adhesion, complement and coagulation cascades and arrhythmogenic right ventricular cardiomyopathy (Table III).

## Discussion

Breast cancer is the most common type of invasive cancer amongst females. In previous studies, gene expression profiling has been used to predict the clinical outcomes of breast cancer (22) and the distant metastasis of lymph-node-negative primary breast cancer (23). In the present study, using the gene expression patterns downloaded from the GEO database, 571 DEGs between breast cancer and normal tissues were identified, including 241 upregulated and 330 downregulated genes. GO analysis identified that several functional terms were statistically enriched by the DEGs, which were associated with cell adhesion, the extracellular region, response to wounding and immune response.

Cell adhesion is a common process in numerous biological processes, including cell-cell and cell-matrix interactions (24). Cell adhesion is mediated by multiple distinct families of receptors targeting cell adhesion to the ECM, as well as cellular growth, differentiation and migration (25). The tumor metastasis process initially requires the disruption of adhesion interaction between tumor and normal cells or the extracellular matrix, resulting in the release of neoplastic cells, followed by enhanced cell adhesion at later time-points (26). E-cadherin (E-cad) is a type of cell adhesion receptor, which modulates intercellular interactions in epithelial tissues (27). The critical role of E-cad in the invasion and metastasis of breast cancer cells has been previously reported (28). E-cad has been found to be significantly accumulated in breast cancer cells, accompanied by enhanced invasion and metastatic potential of tumor cells (28). Recent evidence has indicated that cellular adhesion molecules also possess prognostic significance in patients with breast cancer (29). Therefore, the expression of E-cad, combined with carcinoembryonic antigens, represents a powerful biomarker for predicting the prognosis of breast cancer.

In addition, cell adhesion is considered to be associated with the response to wounding and immune response. Cell migration of monolayers has been found to occur in response to wounding, surrounding the wound and facilitating wound closure (30). The activation of the immune response depends upon the regulation of cell-cell interactions in the immune system and cell adhesion receptor-regulation of the migration of lymphocytes and cell-cell interactions (30). The present study also demonstrated that the overrepresented pathways were associated with cell adhesion, including focal adhesion and ECM-receptor interactions. Therefore, cell adhesion may have a crucial role in mediating breast cancer development.

In order to explore the interactions of the identified DEGs, the PPI network was constructed. Three genes were identified to be significant nodes with maximum degrees, including *FN1, IL6* and *FOS*. In addition, these three genes were found to be significant nodes in subnetwork 1 (Fig. 3) and were involved in cell surface receptor-linked signal transduction, response to wounding and the extracellular region.

*FN1*, also known as encoding fibronectin 1, is an ECM glycoprotein that binds to interleukin (31). *FN1* was found to be involved in cell adhesion and migration, wound healing and host defense (32), which are in accordance with the functions of subnetwork 1 identified in the present study. The expression of *FN1* is directly regulated by micro (mi)RNA-206, which has been demonstrated to be associated with metastatic cancer types, including breast cancer (33,34). miRNA-206 inhibits cell growth in breast cancer by targeting estrogen receptor 1. Furthermore, *FN1* was found to be correlated with the drug-resistance of cancer cells (35). The expression of *FN1* was reported to be significantly accumulated in vincristine-resistant myeloma cells, while other ECM components, including type II collagen α1, were downregulated. The results of the present study indicated that the *FN1* gene was upregulated in the development of breast cancer and that FN1 was a hub protein with a degree score of 39 in the established PPI network. Therefore, the *FN1* gene was found to be a key regulator in breast cancer development.

FOS is a family of transcription factors including c-Fos, FosB and Fra-1 (36). *c-Fos* is a proto-oncogene associated with cellular functions and has been found to be overexpressed in various types of cancer. *c-Fos* functions as a nuclear transcription factor and plays a crucial role in growth factor signaling (37). *c-Fos* is one of the targets for the estrogen receptor (38), and the expression of *c-Fos* is significantly enhanced by the induction of estrogen in breast cancer cells (39). Estrogen sensitizes breast cancer cells to growth factors, which then contribute to tumor growth (37). In addition, previous studies have indicated that proto-oncogenes, including *c-Fos*, are involved in breast cancer cell cycle-associated functions (40). Progestins, as members of the estrogen family, have been shown to enhance *c-Fos* expression resulting in acceleration of the cell cycle progression (41). Furthermore, IL6 is recognized as a pro-inflammatory cytokine that modulates the inflammatory response (42). The role of *IL6* signaling has been widely investigated in the development of various types of cancer, including liver (43), lung (44) and breast cancer (45). A recent study reported that the expression levels of *IL6* and *IL8* in triple-negative breast cancer (TNBC) were associated with cell survival, and that the inhibition of *IL6*/*IL8* signaling was a therapeutic strategy for improving the prognosis of patients with TNBC (45). Therefore, the key role of *FN1, IL6* and *FOS* in breast cancer development has been demonstrated. These nodes may provide promising targets for the treatment of breast cancer in the future.

In conclusion, gene expression profiles were found to be altered during the development and progression of breast cancer. The cell adhesion, extracellular region and immune response were significant functions of the DEGs identified in breast cancer progression. In addition, the *FN1, IL6* and *FOS* genes were found to be involved in breast cancer development. The present study suggested that *FN1, IL6* and *FOS* may be potential targets in the development of treatments for breast cancer. However, further evaluation of their potential applications is required.

## Figures and Tables

**Table I:** GO and pathway enrichment analysis for DEGs.

Category	Term	Count	P-value
Upregulated DEGs			
GOTERM_BP_FAT	GO: 0007155 - cell adhesion	28	3.21×10^−7^
GOTERM_BP_FAT	GO: 0022610 - biological adhesion	28	3.30×10^−7^
GOTERM_BP_FAT	GO: 0009611 - response to wounding	22	4.93×10^−6^
GOTERM_BP_FAT	GO: 0006955 - immune response	21	6.08×10^−4^
GOTERM_BP_FAT	GO: 0042981 - regulation of apoptosis	21	3.74×10^−3^
GOTERM_CC_FAT	GO: 0005576 - extracellular region	68	3.07×10^−11^
GOTERM_CC_FAT	GO: 0044421 - extracellular region part	40	7.01×10^−9^
GOTERM_CC_FAT	GO: 0005578 - proteinaceous extracellular matrix	24	4.22×10^−10^
GOTERM_CC_FAT	GO: 0031012 - extracellular matrix	24	1.83×10^−9^
GOTERM_CC_FAT	GO: 0005615 - extracellular space	22	1.39×10^−3^
GOTERM_MF_FAT	GO: 0005509 - calcium ion binding	24	1.05×10^−3^
GOTERM_MF_FAT	GO: 0030246 - carbohydrate binding	14	4.94×10^−4^
GOTERM_MF_FAT	GO: 0003779 - actin binding	13	7.97×10^−4^
GOTERM_MF_FAT	GO: 0042277 - peptide binding	9	3.95×10^−3^
GOTERM_MF_FAT	GO: 0005201 - extracellular matrix structural constituent	8	9.73×10^−5^
KEGG_PATHWAY	hsa04060: Cytokine-cytokine receptor interaction	12	5.61×10^−3^
KEGG_PATHWAY	hsa04510: Focal adhesion	10	8.09×10^−3^
KEGG_PATHWAY	hsa04512: Extracellular matrix-receptor interaction	9	9.44×10^−5^
Downregulated DEGs			
GOTERM_BP_FAT	GO: 0007166 - cell surface receptor-linked signal transduction	51	4.35×10^−4^
GOTERM_BP_FAT	GO: 0009611 - response to wounding	28	2.88×10^−7^
GOTERM_BP_FAT	GO: 0007155 - cell adhesion	27	1.29×10^−4^
GOTERM_BP_FAT	GO: 0022610 - biological adhesion	27	1.32×10^−4^
GOTERM_BP_FAT	GO: 0010033 - response to organic substance	26	4.93×10^−4^
GOTERM_CC_FAT	GO: 0005886 - plasma membrane	101	3.38×10^−6^
GOTERM_CC_FAT	GO: 0005576 - extracellular region	66	6.96×10^−7^
GOTERM_CC_FAT	GO: 0044459 - plasma membrane part	66	1.64×10^−5^
GOTERM_CC_FAT	GO: 0044421 - extracellular region part	46	1.76×10^−9^
GOTERM_CC_FAT	GO: 0031226 - intrinsic to plasma membrane	37	1.76×10^−3^
GOTERM_MF_FAT	GO: 0030246 - carbohydrate binding	18	1.02×10^−4^
GOTERM_MF_FAT	GO: 0008289 - lipid binding	17	3.87×10^−3^
GOTERM_MF_FAT	GO: 0030247 - polysaccharide binding	10	1.16×10^−3^
GOTERM_MF_FAT	GO: 0001871 - pattern binding	10	1.16×10^−3^
GOTERM_MF_FAT	GO: 0005539 - glycosaminoglycan binding	8	9.36×10^−3^
KEGG_PATHWAY	hsa04360: Axon guidance	8	9.98×10^−3^
KEGG_PATHWAY	hsa04610: Complement and coagulation cascades	7	1.69×10^−3^

Top five GO terms in various categories and significant pathways are listed. Count, number of DEGs enriched in a GO term/pathway; GO, gene ontology; DEGs, differentially expressed genes; MF, molecular function; BP, biological process; CC, cellular component; KEGG, Kyoto Encyclopedia of Genes and Genomes; FAT, function annotation test.

**Table II:** GO and pathway analysis for genes in subnetwork 1.

Category	Term	Count	P-value
GOTERM_BP_FAT	GO: 0007166 - cell surface receptor linked signal transduction	8	2.12×10^−3^
GOTERM_BP_FAT	GO: 0002237 - response to molecule of bacterial origin	7	2.64×10^−10^
GOTERM_BP_FAT	GO: 0009617 - response to bacterium	7	3.51×10^−8^
GOTERM_BP_FAT	GO: 0007610 - behavior	7	6.45×10^−6^
GOTERM_BP_FAT	GO: 0009611 - response to wounding	7	1.30×10^−5^
GOTERM_CC_FAT	GO: 0044421 - extracellular region part	9	3.93×10^−6^
GOTERM_CC_FAT	GO: 0005576 - extracellular region	9	8.38×10^−4^
GOTERM_CC_FAT	GO: 0005615 - extracellular space	8	5.43×10^−6^
KEGG_PATHWAY	hsa05200: Pathways in cancer	7	8.02×10^−5^
KEGG_PATHWAY	hsa04620: Toll-like receptor signaling pathway	4	1.88×10^−3^

Top five GO terms in various categories and significant pathways are listed. Count, number of DEGs enriched in a GO term/pathway; GO, gene ontology; MF, molecular function; BP, biological process; CC, cellular component; KEGG, Kyoto Encyclopedia of Genes and Genomes; FAT, function annotation test.

**Table III:** GO and pathway analysis for genes in subnetwork 2.

Category	Term	Count	P-value
GOTERM_BP_FAT	GO: 0007155 - cell adhesion	13	4.68×10^−12^
GOTERM_BP_FAT	GO: 0022610 - biological adhesion	13	4.76×10^−12^
GOTERM_BP_FAT	GO: 0009611 - response to wounding	7	4.36×10^−05^
GOTERM_BP_FAT	GO: 0042060 - wound healing	5	1.01×10^−04^
GOTERM_BP_FAT	GO: 0001501 - skeletal system development	5	7.15×10^−04^
GOTERM_CC_FAT	GO: 0005576 - extracellular region	15	2.88×10^−08^
GOTERM_CC_FAT	GO: 0044421 - extracellular region part	14	3.97×10^−11^
GOTERM_CC_FAT	GO: 0005578 - proteinaceous extracellular matrix	12	1.29×10^−13^
GOTERM_CC_FAT	GO: 0031012 - extracellular matrix	12	2.95×10^−13^
GOTERM_CC_FAT	GO: 0009986 - cell surface	8	3.97×10^−7^
GOTERM_MF_FAT	GO: 0005509 - calcium ion binding	7	7.77×10^−4^
GOTERM_MF_FAT	GO: 0001948 - glycoprotein binding	6	8.57×10^−10^
GOTERM_MF_FAT	GO: 0005201 - extracellular matrix structural constituent	5	4.00×10^−6^
GOTERM_MF_FAT	GO: 0005539 - glycosaminoglycan binding	5	2.76×10^−5^
GOTERM_MF_FAT	GO: 0001871 - pattern binding	5	4.02×10^−5^
KEGG_PATHWAY	hsa04512: extracellular matrix-receptor interaction	7	3.38×10^−9^
KEGG_PATHWAY	hsa04510: Focal adhesion	6	1.97×10^−5^
KEGG_PATHWAY	hsa04610: Complement and coagulation cascades	3	7.61×10^−3^
KEGG_PATHWAY	hsa05412: Arrhythmogenic right ventricular cardiomyopathy	3	9.18×10^−3^

Top five GO terms in various categories and significant pathways are listed. Count, number of DEGs enriched in a GO term/pathway; GO, gene ontology; MF, molecular function; BP, biological process; CC, cellular component; KEGG, Kyoto Encyclopedia of Genes and Genomes; FAT, function annotation test.
